# AAT resistance-related AC007405.2 and AL354989.1 as novel diagnostic and prognostic markers in prostate cancer

**DOI:** 10.18632/aging.205754

**Published:** 2024-04-19

**Authors:** Yuanzhong Deng, Chunlin Zhang, Haitao Yu, Guo Chen, Xiang Peng, Yang Li, Zhenwei Feng, Wei Shi, Xuesong Bai, Xin Gou, Nian Liu

**Affiliations:** 1 Department of Urology, The First Affiliated Hospital of Chongqing Medical University, Yuzhong, Chongqing 400016, China; 2 Chongqing Key Laboratory of Molecular Oncology and Epigenetics, Yuzhong, Chongqing, China

**Keywords:** prostate cancer, lncRNA, AAT resistance, risk score model, prostate cancer diagnosis

## Abstract

Objective: Prostate cancer (PCa) is the second disease threatening men’s health, and anti-androgen therapy (AAT) is a primary approach for treating this condition. Increasing evidence suggests that long non-coding RNAs (lncRNAs) play crucial roles in the development of PCa and the process of AAT resistance. The objective of this study is to utilize bioinformatics methods to excavate lncRNAs association with AAT resistance and investigate their biological functions.

Methods: AAT resistance-related risk score model (ARR-RSM) was established by multivariate Cox analysis. Paired clinical tissue samples of 36 PCa patients and 42 blood samples from patients with PSA over 4 ng/ml were collected to verify the ARR-RSM. *In vitro*, RT-qPCR, CCK-8 and clone formation assays were displayed to verify the expression and function of AL354989.1 and AC007405.2.

Results: Pearson correlation analysis identified 996 lncRNAs were associated with AAT resistance (ARR-LncRs). ARR-RSM was established using multivariate Cox regression analysis, and PCa patients were divided into high-risk and low-risk groups. High-risk patients showed increased expression of AL354989.1 and AC007405.2 had poorer prognoses. The high-risk score correlated with advanced T-stage and *N*-stage. The AUC of ARR-RSM outperformed tPSA in diagnosing PCa. Silencing of AC007405.2 and AL354989.1 inhibited PCa cells proliferation and AAT resistance.

Conclusions: In this study, we have discovered the clinical significance of AC007405.2 and AL354989.1 in predicting the prognosis and diagnosing PCa patients. Furthermore, we have confirmed their correlation with various clinical features. These findings provide potential targets for PCa treatment and a novel diagnostic and predictive indicator for precise PCa diagnosis.

## INTRODUCTION

Prostate cancer (PCa) is the second prevalent malignancy and the fifth leading cause of cancer death among men worldwide, with more than 1.4 million new cases of PCa and 375, 000 deaths each year [1, 2]. Since most PCa patients are diagnosed with localized disease, the survival rate can be closed to 100% through radical prostatectomy and radiation therapy [3–5]. Unfortunately, nearly 10% of PCa patients in underdeveloped regions will develop metastatic disease, with a 5-year survival rate dropping to around 30% [6]. With rapid aging process, PCa is becoming a major urinary malignancy affecting men’s health.

Prostate-specific antigen (PSA) is the most widely recognized prostate-specific biomarker for PCa [7]. However, its sensitivity and specificity for diagnosing PCa are limited [8]. Besides PCa, elevated levels of total PSA (tPSA) can also occur due to benign prostate conditions such as chronic prostatitis, benign prostatic hyperplasia, urinary tract infections, prostate manipulation, and ejaculation. Due to these limitations, it is challenging to establish a universally applicable threshold for diagnosing PCa. There is an urgent need in clinical practice for biomarkers that can accurately diagnose PCa. Prostate biopsy remains the gold standard for confirming PCa [9]. Patients with tPSA >10 ng/mL require PCa biopsy, but among men who undergo biopsy due to elevated tPSA levels, only about 25–30% are found to have cancer, while the majority experience false-positive results and undergo unnecessary biopsies [10, 11]. There is still no definitive marker to determine whether PCa patients with tPSA levels between 4–10 ng/mL (PSA gray zone) require biopsy [12]. Therefore, there is a clinical need for biomarkers that can accurately guide PCa biopsy decisions.

Due to PCa being an androgen-dependent cancer, androgen deprivation therapy has become an essential treatment option for locally advanced and metastatic PCa, as well as the basis for a variety of novel combination therapy regimens, and is often required throughout the patient's subsequent treatment [6, 13]. In addition, anti-androgen therapy drugs (AAT) such as enzalutamide (ENZ), bicalutamide (BIC) and apalutamide (ARN), competitively blocking androgen binding to androgen receptors (AR) on prostate cells, are also widely used in advanced PCa [14, 15]. In the initial stage of AAT treatment, most tumors can be significantly inhibited, showing ideal efficacy and decreased PSA [16]. But eventually, as the disease progresses, PCa will recur as castration-resistant prostate cancer (CRPC) [17, 18]. Therefore, identifying remarkable biomarkers related to AAT resistance is advantageous to treatment of PCa and helpful to find novel and viable targets.

Long non-coding RNAs (lncRNAs) are RNA transcripts more than 200 nucleotides in length, with no or limited protein-coding potential and limited evolutionary conservation, but particularly significant on various biological functions in cancer cell, including epigenetic regulation, DNA damage repair and cell-cycle regulation [19–22]. Numerous studies have shown that expression of lncRNAs undergoes dysregulation in PCa, which leads to recurrence, progression and metastasis [23, 24]. lncRNA-PCAT1 is positively linked to CRPC progression by perturbing the PHLPP/FKBP51/IKKα complex and activating AKT and NF-κB signaling [24]. High m6A level of lncRNA-NEAT1-1 is related to promote bone metastasis of PCa, and overexpression of NEAT1-1 actually induces PCa metastasis to lung and bone [25]. Therefore, lncRNAs are also frequently serving as effective biomarkers for assessing PCa prognosis. However, the effect of AAT resistance mediated alteration of lncRNAs on PCa prognosis and diagnosis remain poorly understood, which is worth further study and discovery.

Here, in order to investigate the function of AAT resistance-related lncRNAs (ARR-LncRs) and establish a precise AAT resistance-related risk score model for diagnosing PCa (ARR-RSM), we utilized PCa transcriptional data and clinical data from TCGA database. PSA screening data and serum qPCR data from Sichuan and Chongqing were used to compare the diagnostic performance between ARR-RSM and PSA. Our results will provide reliable evidence for the diagnosis of PCa and will offer personalized AAT treatment options for PCa patients.

## MATERIALS AND METHODS

### Clinical blood and tissue sample

From December 2020 to March 2023, 36 PCa adjacent-normal tissues and tumor tissues from patients who underwent tumor excision in the First Affiliated Hospital of Chongqing Medical University were collected, and immediately stored in liquid nitrogen until RNA extraction. From December 2020 to December 2022, during the implementation of the project on PCa screening and intervention in Sichuan and Chongqing, we collected blood samples and PSA screening results from 570 patients in the First Affiliated Hospital of Chongqing Medical University.

### PCa screening subjects

The screening subjects were from a total of 47 hospitals in Chongqing and Sichuan. The population standard for PCa screening includes: (1) males in good physical condition with a life expectancy of more than 10 years; (2) males over 50 years of age; males over 45 years of age with a family history of PCa; (3) high-risk individuals including males over 50 years of age; males over 45 years of age with a family history of PCa; males over 40 years of age with a baseline PSA >1 μg/L.

### Cell culture and treatment

Normal human prostate epithelial cells (RWPE-1) and PCa cells (LNCaP) were purchased from Shanghai Zhong Qiao Xin Zhou Biotechnology Co., Ltd., (Shanghai, China). LNCaP cells were cultured in RPMI-1640 basal medium (Gibco, USA) supplemented with 10% fetal bovine serum (Procell, Wuhan, China), 100 U/ml penicillin (Beyotime, Beijing, China) and 0.1 mg/ml streptomycin (Beyotime, Beijing, China) and incubated in a humidified cell incubator with 5% CO_2_ at 37°C. LNCaP cells were treated with ENZ, BIC, and ARN solution (MCE, Shanghai, China) for 24 h to simulate drug therapy, and then collected for further experiments. RWPE-1 cells were cultured in special medium (ZQ-1303) and purchased from Shanghai Zhong Qiao Xin Zhou Biotechnology Co., Ltd. (China).

### Cell transfection

SiR-AL354989.1 and siR-AC007405.2 were transfected in LNCaP to silence the expressions of AL354989.1 and AC007405.2 (Tsingke, Beijing, China). The sequences used were: siR-AL354989.1-1 (sense: 5′-GGAAGCUUAUGCAAUAGGA(dT)(dT)-3′, antisense: 5′-UCCUAUUGCAUAAGCUUCC(dT)(dT)-3′); siR-AL354989.1-2 (sense: 5′-GAGGAGGACUGCUAGUCAA(dT)(dT)-3′, antisense: 5′-UUGACUAGCAGUCCUCCUC (dT)(dT)-3′); siR-AC007405.2-1 (sense: 5′-CAGAUCUGUGACCCUGCGA(dT)(dT)-3′, antisense: 5′-UCGCAGGGUCACAGAUCUG(dT)(dT)-3′); siR-AC007405.2-2 (sense: 5′-GGAUUGUUGUAACCGCAAA(dT)(dT)-3′, antisense: 5′-UUUGCGGUUACAACAAUCC(dT)(dT)-3′). For transient transfection of LNCaP, 1 × 10^5^ cells were cultured in T25 flasks. According to the manufacturer’s instruction, LNCaP cells were transfected with 10 μL siRNAs (20 μM) and 5 μL Lipofectamine 3000 (Invitrogen, USA) for 6 h in non-FBS RPMI-1640 basal medium, and then cells were cultured in completed RPMI-1640 medium for 48 h. RT-qPCR was used to verify the silencing efficiencies of siR-AL354989.1 and siR-AC007405.2.

### RNA isolation and RT-qPCR

The TRIzol reagent (Abclonal, Wuhan, China) was used to extract total RNA from cells and clinical tissues according to the manufacturer’s instruction. Total RNA was extracted from whole blood using the Whole Blood RNA Isolation Kit (Simgen, Hangzhou, China). 1 μg RNA was reverse transcribed using the PrimeScript RT-qPCR kit (Abclonal, China). RT-qPCR was performed using the SYBR(R) Prime-Script RT-qPCR kit (Abclonal, Wuhan, China) on an ABI 7500 real-time PCR system (Applied Biosystems, USA). The 2^−ΔΔCt^ method was used to calculate the Ct values. The lncRNA values were normalized to the expression levels of β-actin. The primer sequences of lncRNAs and β-actin are shown in Table 1.

**Table 1:** List of primer sequence used in this study.

**β-actin**	Forward	CCTTCCTGGGCATGGAGTC
	Reverse	TGATCTTCATTGTGCTGGGTG
**AC007405.2**	Forward	CTCCTCTACCTAGCCCCCTG
	Reverse	GGGGAAAAGCACTGAGGCAA
**AL354989.1**	Forward	GGCCGAAGACCATAAGTGCT
	Reverse	AGCTTCCTGAGAGGCTCCTT

Abbreviations: F primer: forward primer; R primer: reverse primer.

### Colony formation assay

LNCaP cells under different treatments were seeded at 1000 cells per well in a 6-well plate and incubated at 37°C with 5% CO_2_ for 10 days until visible colonies formed. After incubation, the cultured cells were washed three times with PBS and fixed with a solution of 4% paraformaldehyde and stained with 0.1% crystal violet for 20 minutes. The number of colonies was counted using ImageJ software after capturing image.

### Cell counting kit-8 (CCK-8)

CCK-8 was used for the cells’ proliferation assay by following the manufacturer’s protocol. 2 × 10^3^ LNCaP cells per well were placed in a 96-well plate with 200 μL completed RPMI-1640 medium and cultured in an incubator at 5% CO_2_ and 37°C. The drug sensitivity test of LNCaP cells was also detected by CCK-8. After treating with enzalutamide, bicalutamide and apalutamide for 48 h, 2 × 10^3^ treated LNCaP cells were cultured in a 96-well plate and cultured in an incubator at 5% CO_2_ and 37°C. CCK-8 reagent 10 μL (Dojindo, Kumamoto, Japan) was added to each well at 0 h, 6 h, 24 h, 48 h, 72 h and incubated at 37°C for 1.5 h. The absorbance was measured at 450 nm by microplate reader.

### PCa transcriptome data of TCGA preprocessing

Transcriptome RNA sequencing and clinical data of 492 PCa tumor tissues and 51 non-tumor samples were downloaded from TCGA data portal (https://portal.gdc.cancer.gov/). PCa patients with an overall survival (OS) ≤30 days were excluded from the analysis to eliminate the influence of unpredictable factors such as hemorrhage and infection on OS. The original data of PCa patients were collected for subsequent analysis. The transcriptome RNA sequencing results and clinical data of PCa patients were integrated into a matrix file using a merge script written in the Perl programming language (http://www.perl.org/).

### Survival-related ARR-LncRs (sARR-LncRs)

AAT resistance-related genes were obtained from the Gene Expression Omnibus database (GSE211781). Pearson correlation analysis was performed to examine the correlation between AAT resistance-related gene and the expressions of lncRNAs in PCa patients. ARR-LncRs were identified based on the criteria of |r| > 0.7 and *P* < 0.05 (Supplementary Table 1). Subsequently, sARR-LncRs were selected through univariate Cox analysis using the survival package in the R software (*P* < 0.001). Finally, the sARR-LncRs were further categorized as either harmful or protective based on their hazard ratio (HR).

### AAT resistance-related risk score model (ARR-RSM)

The ARR-RSM was developed using the selected sARR-LncRs through multivariate Cox regression analysis (Supplementary Table 2). The risk score for each PCa patient was calculated by multiplying the expression levels of the sARR-LncRs by their corresponding Cox regression coefficients. The formula used for calculation was as follows: ((Expression level of AL354989.1) × (2.037602888)) + ((Expression level of AC007405.2) × (1.765286435)). Based on the median score, PCa patients were categorized into either the high-risk group or the low-risk group.

### Bioinformatics analysis

We evaluated the survival rate of patients in the ARR-RSM using the survival package. Receiver operating characteristic (ROC) curves were generated, and the area under the curve (AUC) was calculated using the survival ROC package to assess the accuracy of the ARR-RSM. Multivariate and univariate Cox regression analysis was conducted to confirm the independent prognostic factors for PCa patients. To predict the survival rate of PCa patients, we utilized the nomogram provided by the rms package. Kyoto Encyclopedia of Genes and Genomes (KEGG) in gene set enrichment analysis (GSEA) was employed to identify the underlying pathways associated with ARR-RSM.

### Statistical analysis

Statistical analysis was performed using SPSS version 21.0 (SPSS, USA) and GraphPad Prism 8.0 (GraphPad Software Inc., USA). The data are presented as means ± SD. The correlation between the expression levels of sARR-LncRs and clinical features was evaluated using Fisher’s exact probability method. Student’s *t*-test, ANOVA, and post-hoc tests were employed for comparing differences between two or more groups. *P* < 0.05 was considered statistically significant.

### Availability of data and materials

Authors can provide all data sets analyzed during the study on reasonable requirements.

## RESULTS

### Establishing the ARR-RSM by sARR-LncRs

**Figure 1:**
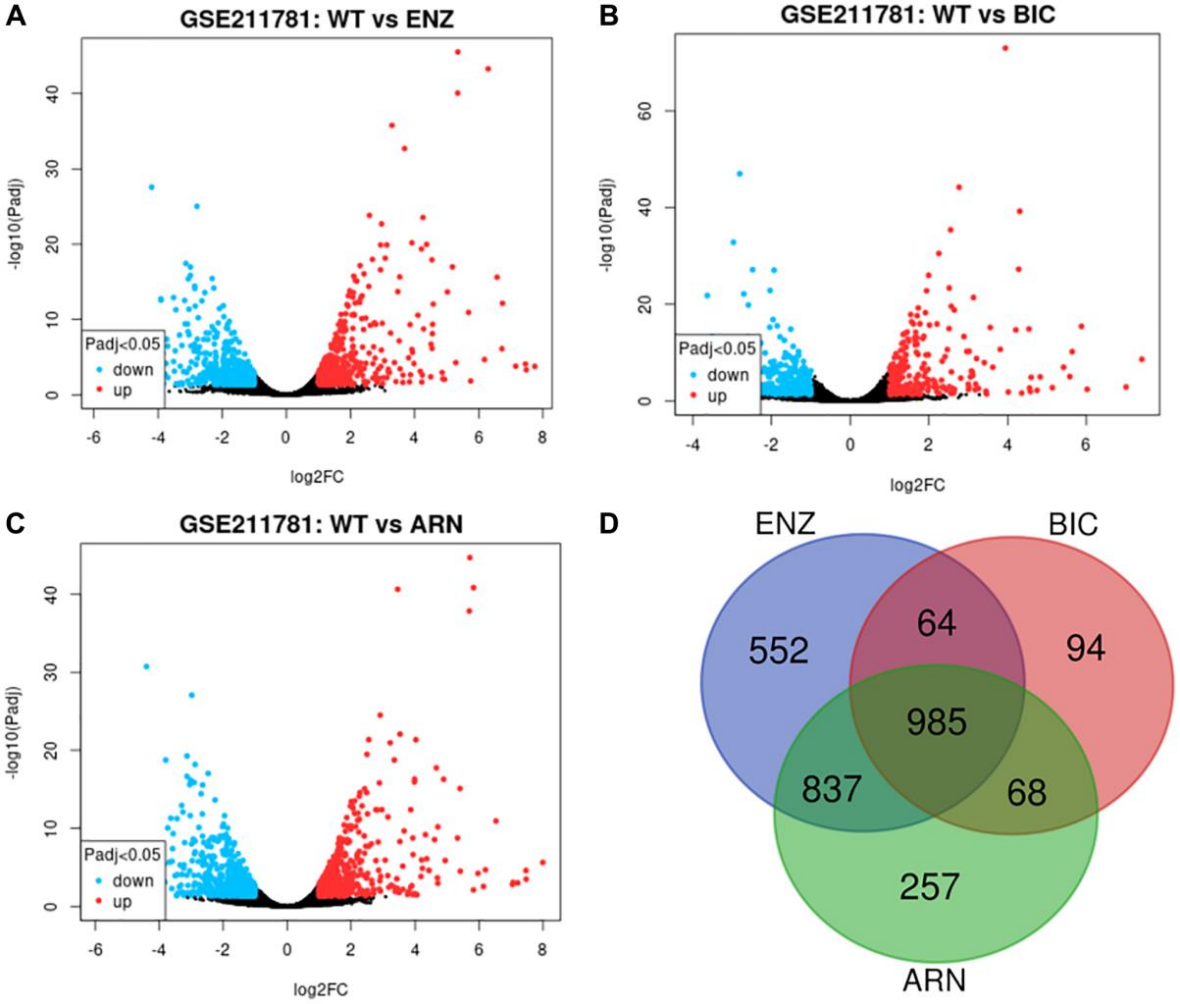
**AAT resistance-related gene selected from GSE211781 dataset.** The differentially expressed genes between ENZ resistance (**A**), BIC resistance (**B**), ARN resistance (**C**) and WT LNCap cell, log_2_|FC|≥1, *P* < 0.05. Venn diagram showed the intersection genes of these differentially expressed genes (**D**).

**Figure 2:**
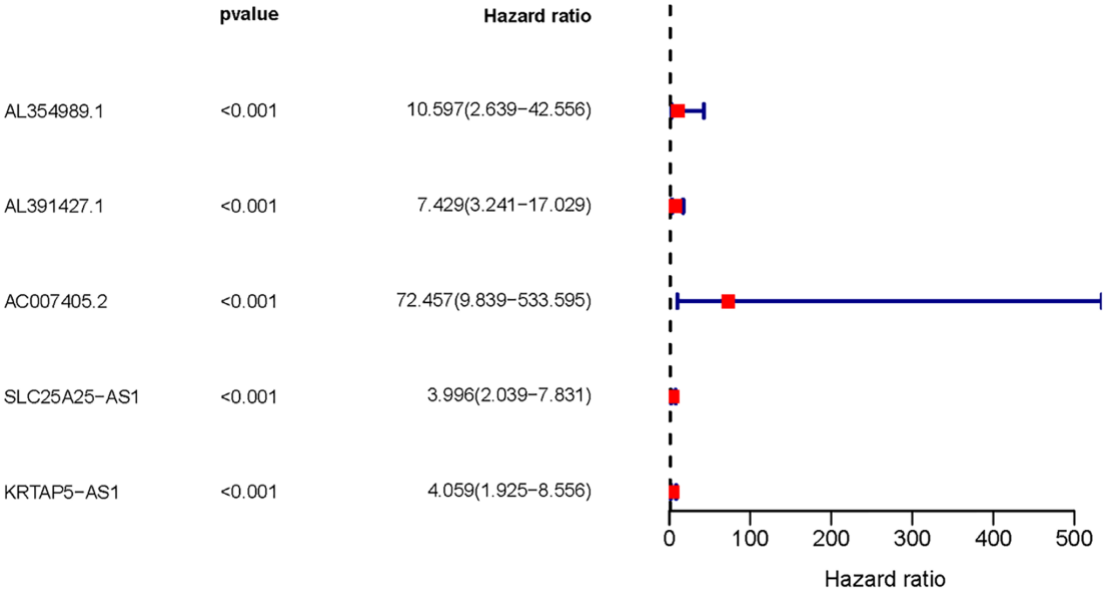
**The forest plot of sARR-LncRs.** The forest plot showed the hazard ratios of AL354989.1, AL391427.1, AC007405.2, SLC25A25-AS1, and KRTAP5-AS1.

**Figure 3:**
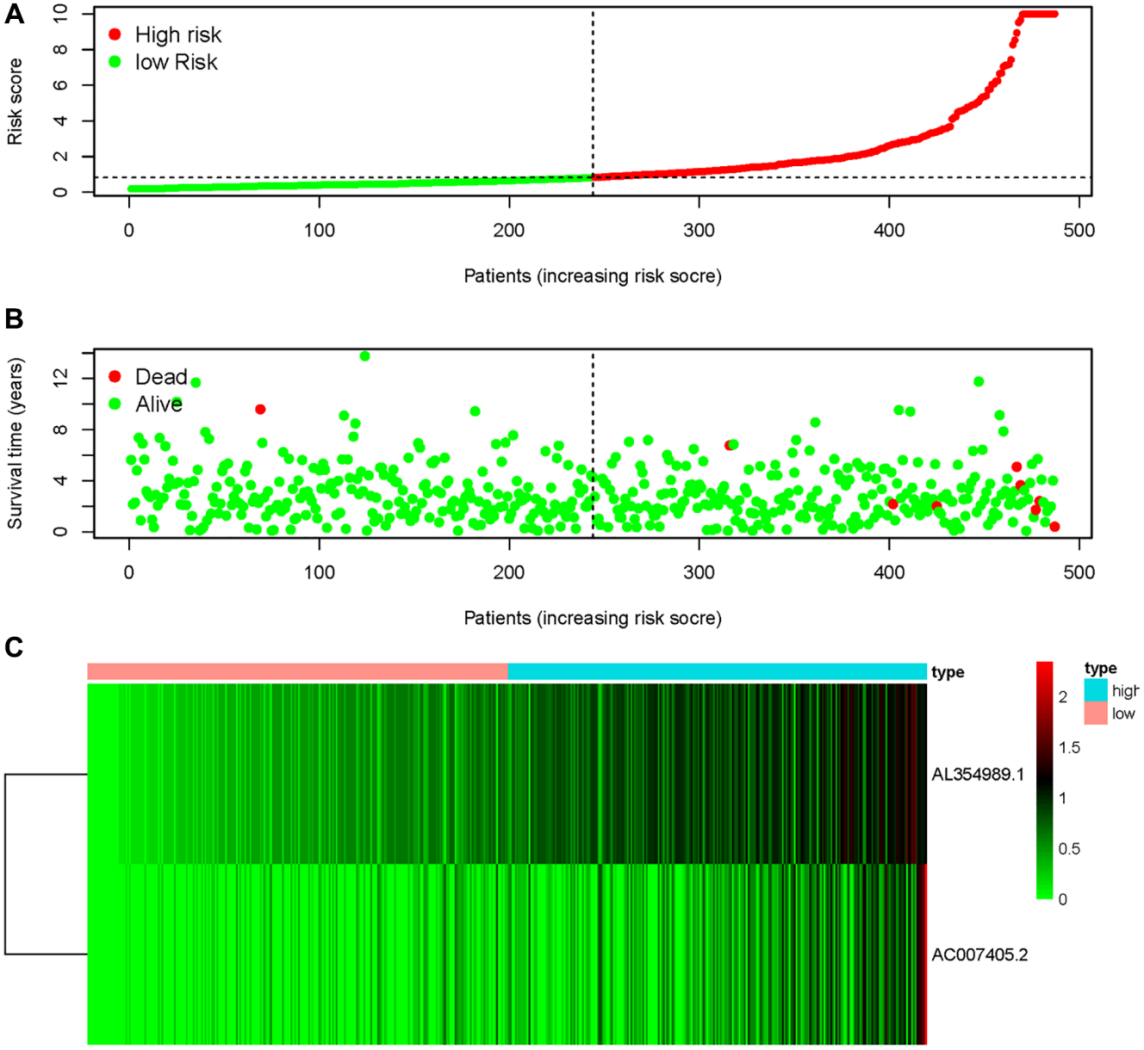
**AAT resistance-related risk score model.** (**A**) The risk score of ARR-RSM and the high-risk group were located on the right by the median risk score. (**B**) The survival status of PCa patients with different risk score. (**C**) The heatmap of expression of AL354989.1 and AC007405.2 in the ARR-RSM.

**Figure 4:**
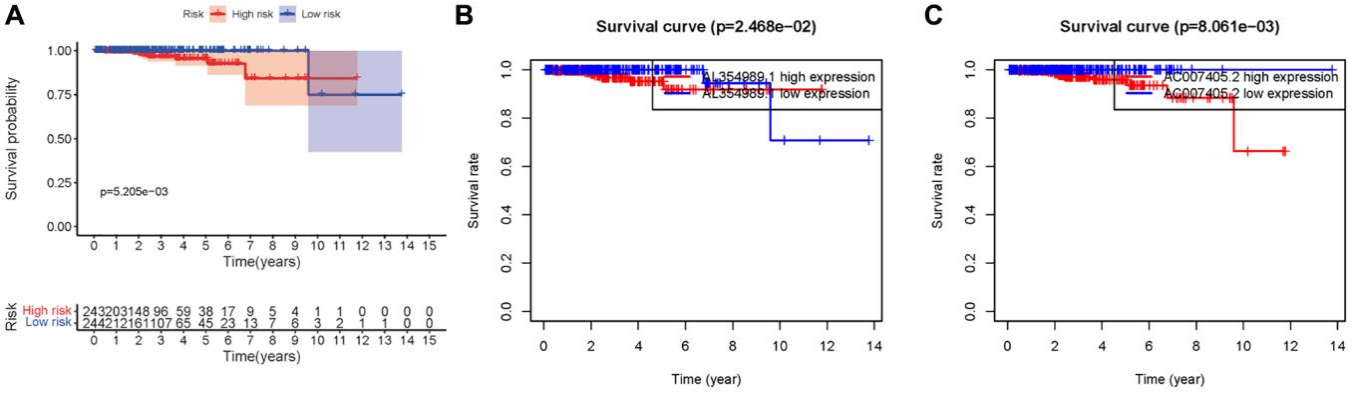
**Kaplan-Meier survival curve of ARR-RSM.** (**A**) Kaplan-Meier survival curve of the high-risk group and low-risk group in ARR-RSM. (**B**, **C**) The survival curves of sARR-LncRs (AL354989.1 and AC007405.2).

We obtained transcriptome RNA-sequencing data and clinical data of PCa patients from TCGA database. After that, we utilized the GSE211781 of GEO Database to screen out 985 AAT resistance-related genes (Figure 1). We found that there were 996 lncRNAs associated with AAT resistance by Pearson correlation analysis (|r| > 0.7 and *P* < 0.05). In parallel, to investigate the relationship between ARR-LncRs and the prognosis of PCa patients, univariate Cox regression analysis was performed and the detailed results were illustrated in the forest map (Figure 2), in which AL354989.1, AL391427.1, SLC25A25-AS1, KRTAP5-AS1 and AC007405.2 were regarded as a remarkable deleterious factor. Next, we established the ARR-RSM by employing multivariate Cox regression analysis. PCa patients were divided into the high-risk and low-risk groups based on the median risk score (Figure 3A). The mortality of PCa patients was positively correlated with the increase of risk score (Figure 3B). In the high-risk group, the expression of AL354989.1 and AC007405.2 were remarkably increased (Figure 3C). Moreover, being revealed by Kaplan-Meier survival curve, PCa patients in the high-risk group showed poorer prognoses (Figure 4A). The high expressions of AL354989.1 and AC007405.2 were significantly related with PCa patients’ poor prognosis (Figure 4B, 4C).

### Clinical correlation of sARR-LncRs and potential mechanism of ARR-RSM

**Figure 5:**
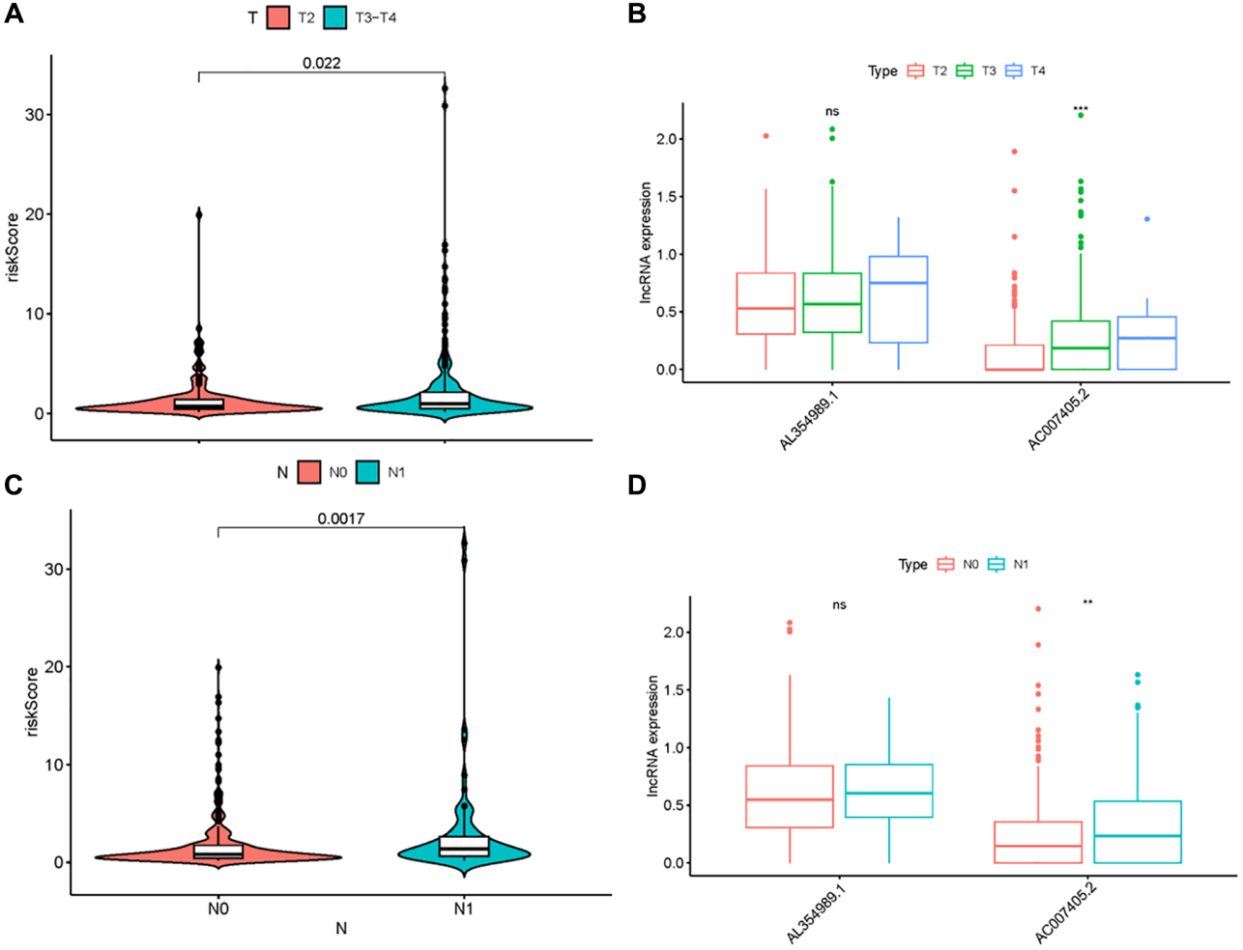
**Clinical correlation analysis of ARR-RSM, AL354989.1 and AC007405.2.** The relations between the expression levels of risk score, AL354989.1 and AC007405.2 with T-stage (**A**, **B**) and *N*-stage (**C**, **D**).

**Figure 6:**
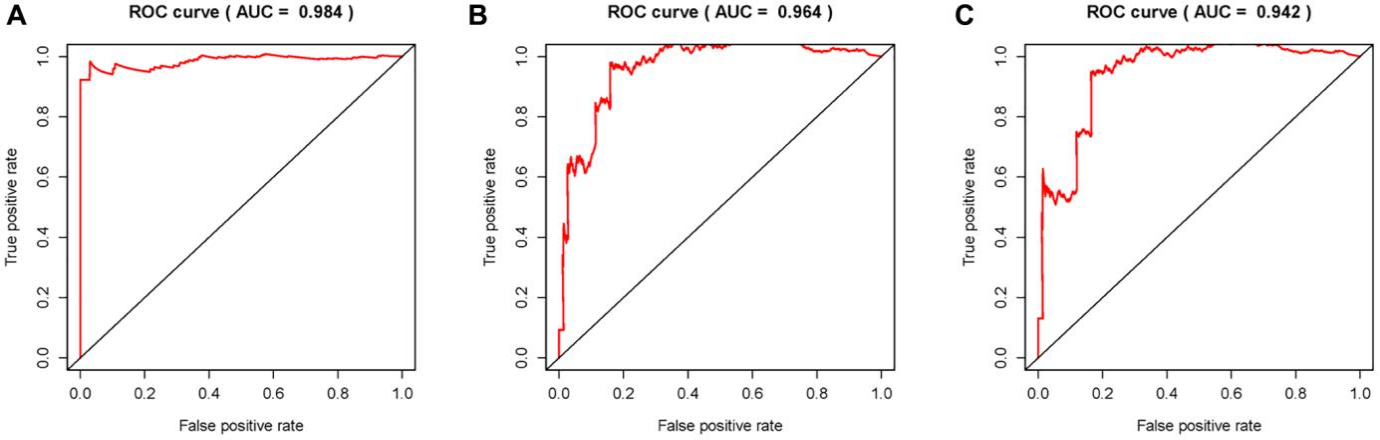
**Receiver operating characteristic (ROC) curves.** Area under curves (AUCs) of 1-, 3- and 5-year ARR-RSM and clinical features. The 1-, 3- and 5-year AUCs’ values of ARR-RSM were 0.984 (**A**), 0.964 (**B**) and 0.942 (**C**) respectively.

**Figure 7:**
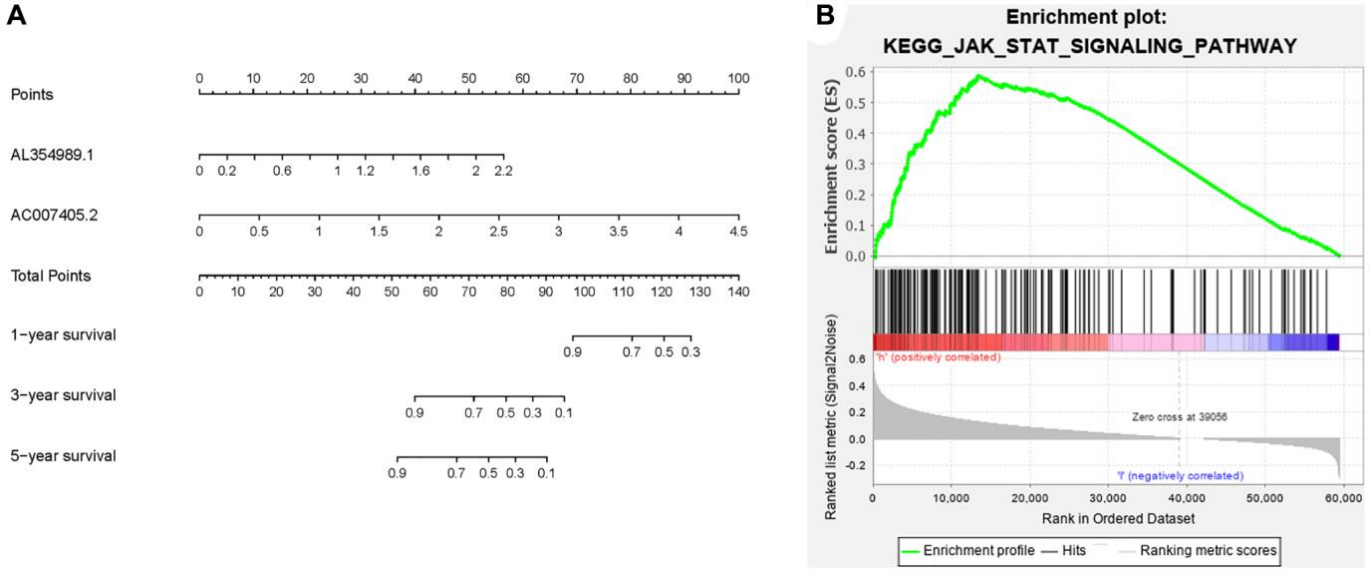
**Clinical application of ARR-RSM and underlying pathway.** (**A**) The nomogram of ARR-RSM could predict 1-, 3- and 5-year survival probabilities of PCa patients by detecting the expressions of sARR-LncRs. (**B**) JAK-STAT pathway was the most enriched pathway in ARR-RSM by KEGG analysis.

Given that sARR-LncRs may suggest a poor prognosis, we used ggpubr package to investigate the correlation between ARR-RSM, sARR-LncRs, and clinicopathologic features of PCa patients, especially T-stage and *N*-stage. The high-risk score correlated with the advanced T-stage and *N*-stage (Figure 5A, 5C). The expressions of AC007405.2 were significantly high in advanced T-stage and *N*-stage, compared to early T-stage and *N*-stage (Figure 5B, 5D). Univariate and multivariate analysis was performed to explore the potential independent risk factor of PCa patients, revealing that risk score was the only independent risk factor of PCa patients (Table 2). Moreover, we focused on the accuracy of ARR-RSM. The AUCs for ROC curves of ARR-RSM and clinical characters were figured out, which showed that the AUCs of 1-, 3- and 5-year risk scores were 0.984, 0.964 and 0.942, respectively (Figure 6A–6C). Altogether these data suggested that the most accurate and valuable independent risk factor of PCa patients was the ARR-RSM. Following that, ranging from 0 to 100, the points of ARR-RSM were normalized. Drawing the ARR-LncRs expressions line between the total points axis and each prognosis axis, the 1-year, 3-year and 5-year survival probabilities were calculated (Figure 7A). These data therefore indicated that, for predicting the prognoses of PCa patients, ARR-RSM was able to be employed as a risk factor. To further investigate the potential mechanism of ARR-RSM, we then performed the Kyoto Encyclopedia of Genes and Genomes (KEGG) pathway analysis of GSEA. As exhibited in Figure 7B, JAK-STAT signaling pathway was the most closely related one to the high-risk group. These results encourage us to further verify the function of sARR-LncRs by basic experiment and examine the association between ARR-RSM and PCa diagnosis and prognosis in clinical samples.

**Table 2:** Univariate and multivariate Cox analysis of PCa patients.

**Variables**	**Univariate analysis**				**Multivariate analysis**			
	**HR**	**HR 95% low**	**HR 95% high**	***P*-value**	**HR**	**HR 95% low**	**HR 95% high**	***P*-value**
**Age**	1.060496	0.953979	1.178907	0.276770	1.070722	0.960997	1.192975	0.215432
**T-stage**	1.961986	0.453625	8.485833	0.367054	1.180598	0.227758	6.119700	0.843240
***N*-stage**	3.436471	0.761458	15.50883	0.108378	3.965234	0.753114	20.87741	0.104074
**Risk score**	1.030819	0.997738	1.064998	0.038166	1.035487	1.000663	1.071524	0.045719

Abbreviation: HR: Hazard Ratio.

### PCa screening data

A total of 16,746 men in Chongqing and Sichuan underwent PSA screening. The overall median age was 64.0 years (ranging from 45 to 89 years), with an average age of 63.63 years. The median total PSA level was 2.00 ng/ml (ranging from 0.01 to 102.00 ng/ml), with an average PSA level of 2.45 ng/ml. Among them, there were 1,005 cases (6%) with abnormal PSA levels. The median PSA level for those with abnormal PSA levels was 6.10 ng/ml (ranging from 4.0 to 102 ng/ml), with an average level of 10.55 ng/ml (Supplementary Figure 1A). Among the 570 men tested at the First Affiliated Hospital of Chongqing Medical University, 52 cases (9.12%) had abnormal PSA levels (Supplementary Figure 1B). Among the men with abnormal PSA levels at the First Affiliated Hospital of Chongqing Medical University, 42 cases agreed to undergo prostate biopsy, resulting in a compliance rate of 80.77% (42/52). Among them, 11 cases were diagnosed with PCa, resulting in a positivity rate of 26.19% (11/42) (Supplementary Figure 1C).

### AC007405.2 and AL354989.1 obtain remarkable clinical significance and relevance

**Figure 8:**
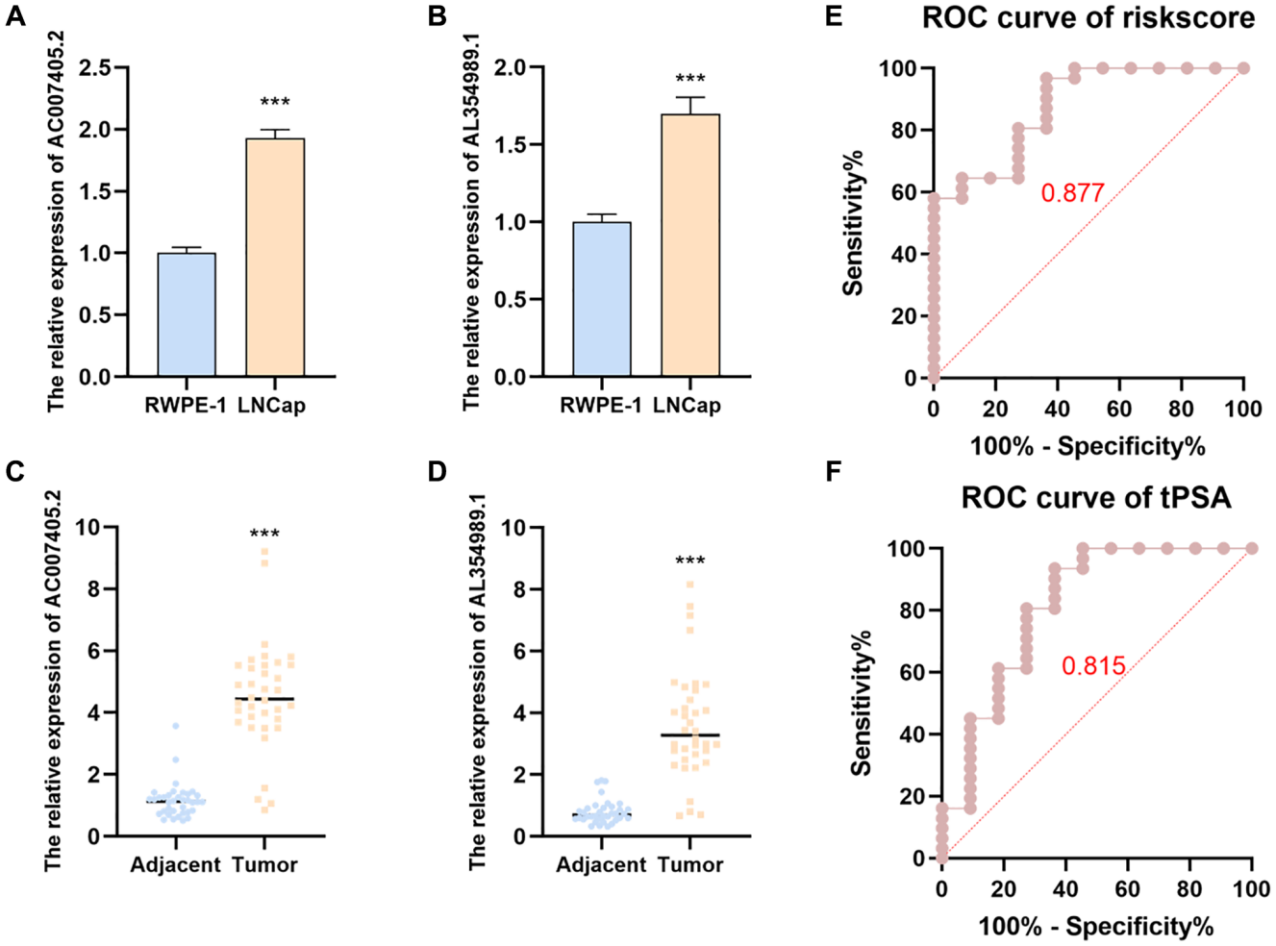
**The expression levels and predicted function of AL354989.1 and AC007405.2 in PCa cell lines and clinical samples.** AL354989.1 and AC007405.2 expressed higher in LNCaP (**A**, **B**) cells and PCa tissues (**C**, **D**) than that in prostate normal tissue and RWPE-1 cells, respectively. ROC curves were generated to predict the occurrence of PCa in patients based on the expression levels of AL354989.1 and AC007405.2 in blood samples (**E**), as well as tPSA values (**F**).

In order to investigate the expression levels of AC007405.2 and AL354989.1 in PCa, we respectively detected AC007405.2 and AL354989.1 in LNCaP cells and then compared them with normal prostate cells RWPE-1. Expectedly, the expression levels of AC007405.2 and AL354989.1 in LNCaP were significantly higher than RWPE-1 (Figure 8A–8B). The same results were confirmed by comparing 36 paired PCa tissues with adjacent normal tissues. The expressions of AC007405.2 and AL354989.1 in PCa tissues were remarkable higher than that in adjacent normal tissues (Figure 8C, 8D). We performed RT-qPCR to measure the expression levels of AC007405.2 and AL354989.1 in the blood samples of patients with tPSA >4 ng/ml. Based on the prostate biopsy results of patients (Supplementary Table 3) we generated ROC curves using the risk score. As shown in Figure 8E, 8F, the accuracy of diagnosing PCa using the risk score (0.877) is significantly higher than using tPSA (0.815) These findings indicate that by measuring the expression levels of AC007405.2 and AL354989.1 in patients’ blood and calculating the risk score, it is possible to more accurately predict whether a patient has PCa. This provides a novel and feasible diagnostic and predictive indicator for precise PCa diagnosis in clinical settings.

### AC007405.2 and AL354989.1 prominently facilitated the proliferation and AAT resistance of PCa cells

**Figure 9:**
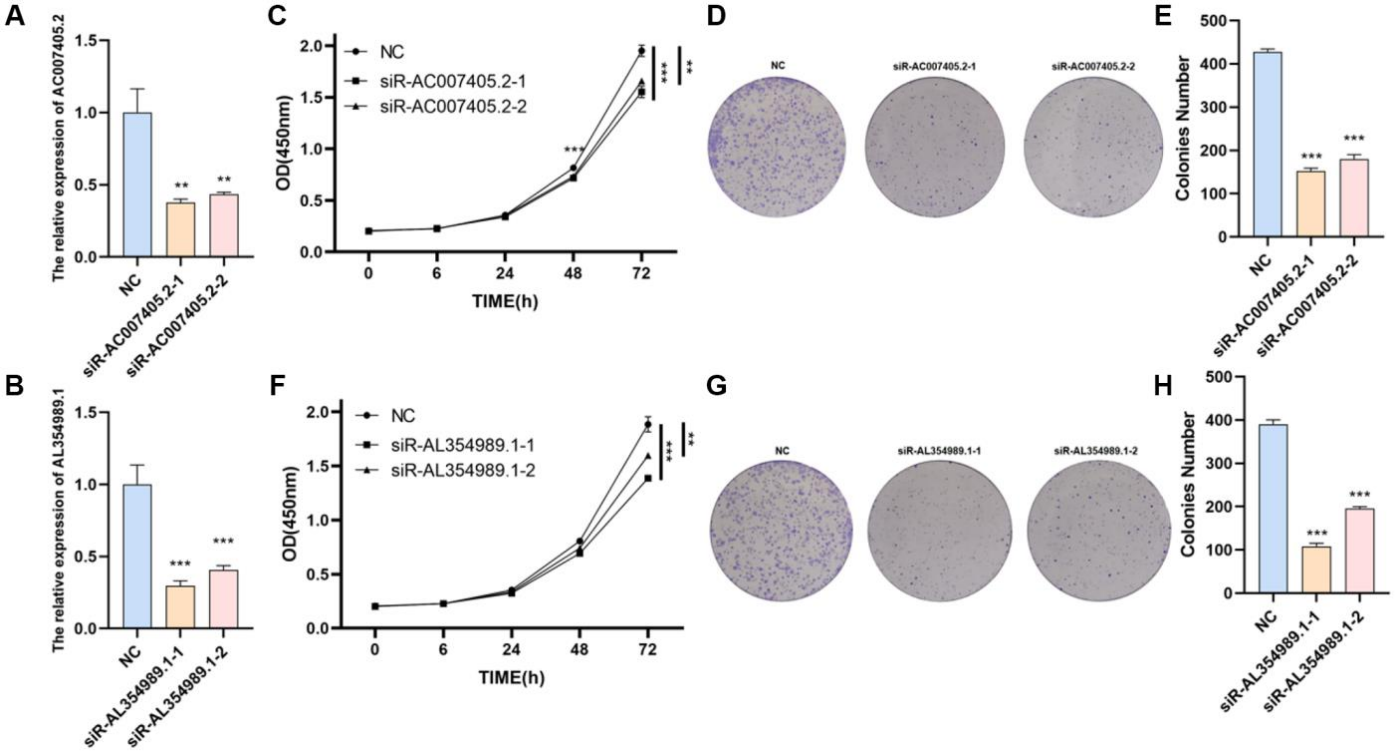
**The effects of AL354989.1 and AC007405.2 on proliferation in LNCaP cells.** The silencing efficiency of siR-AC007405.2 (**A**) and siR-AL354989.1 (**B**). The CCK-8 and clone formation assays showed that silencing of AC007405.2 (**C**–**E**) and AL354989.1 (**F**–**H**) could decrease the proliferation ability of LNCaP cells.

**Figure 10:**
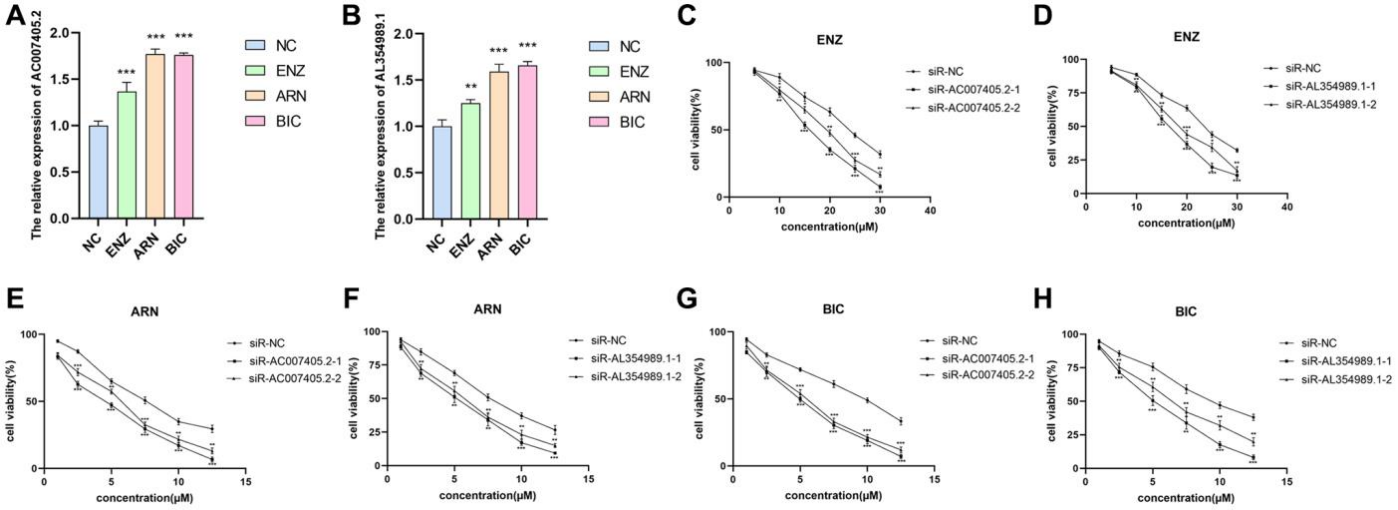
**The effects of AL354989.1 and AC007405.2 on AAT resistance in LNCaP cells.** The expression levels of AC007405.2 (**A**) and AL354989.1 (**B**) were increased with the treatment of ENZ, ARN and BIC, respectively. The drug sensitivity curves of siR-AC007405.2 and siR-AL354989.1 in LNCaP cells under ENZ (**C**, **D**), ARN (**E**, **F**) and BIC (**G**, **H**) treatment, separately.

We first performed siRNAs to knockdown the expression of AC007405.2 (Figure 9A) and AL354989.1 (Figure 9B) and investigated the roles of AC007405.2 and AL354989.1 in proliferation of PCa. The results revealed that both siR-AC007405.2 (Figure 9C–9E) and siR-AL354989.1 (Figure 9F–9H) significantly inhibited the proliferation capacity of LNCaP cells. In order to investigate the role of AC007405.2 and AL354989.1 in the process of AAT resistance, we treated LNCaP cells with ENZ, ARN and BIC, respectively. We then detected the expression levels of AC007405.2 and AL354989.1 by RT-qPCR and results showed that AC007405.2 and AL354989.1 were increased by AAT treatment (Figure 10A, 10B). Following that, we silenced AC007405.2 and AL354989.1 by siRNAs and treated with different concentrations of ENZ, ARN, and BIC separately. The results showed that silencing AC007405.2 and AL354989.1 significantly decreased the relative cell activity in the treatments of ENZ (Figure 10C, 10D), ARN (Figure 10E, 10F), and BIC (Figure 10G, 10H). Herein, these results will provide predictions for the clinical efficacy of AAT treatment in PCa and offer novel potential targets for targeted therapy in PCa.

## DISCUSSION

PCa is a prevalent male cancer globally and is increasingly recognized as a significant health concern for middle-aged and elderly men [26]. Timely screening, early diagnosis, and targeted treatment for high-risk individuals are crucial strategies for enhancing the survival rate of PCa [10]. Since the 1980s, PSA combined with digital rectal examination (DRE) has been widely used as an early screening method for PCa, which has significantly improved the early diagnosis rate of PCa. Nevertheless, many other factors, such as prostatitis, benign prostatic hyperplasia, and cycling can cause elevated PSA levels, leading to a high false-positive rate. Furthermore, there exists a gray zone, tPSA levels between 4–10 ng/mL, for prostate biopsy. These false-positive PSA results can lead to patients undergoing unnecessary invasive prostate biopsies [27]. AAT plays a mainstay role in prostate anti-AR therapy. Most patients develop resistance to anti-androgen therapy during long-term treatment, leading to the malignant progression of PCa [28]. Therefore, it is imperative to identify non-invasive molecular biomarkers that can accurately diagnose PCa and evaluate the prognosis of PCa patients, while also providing potential molecular targets for enhancing the therapeutic effect of AAT in PCa.

In response to the current bottleneck in PCa diagnosis and treatment, an increasing number of researchers are beginning to explore molecular biomarkers for diagnosis and treatment. Kuci Emruli et al. conducted a study in which they measured 157 analytes in 363 serum samples from healthy individuals, patients with non-metastatic PCa, and patients with metastatic PCa. They used a recombinant antibody microarray technique. The researchers identified a signature consisting of 69 proteins that could differentiate metastatic PCa patients from healthy controls [29]. The predictive model based on ARG developed by the Hu Dai-Xing team is a reliable prognostic and predictive tool for overall survival and disease-free survival in PCa patients [30]. However, there is still limited research on the predictive role of lncRNAs in the prognosis of PCa patients. In our study, we utilized multivariate Cox regression analysis to construct an ARR-RSM based on the expression levels of AC007405.2 and AL354989.1. This model accurately predicts the prognosis of PCa patients, with ROC values of 0.984, 0.964, and 0.942 for 1, 3, and 5 years, respectively. Its accuracy is significantly higher than risk models constructed by other researchers. Furthermore, we found a significant association between our ARR-RSM and clinical T-stage and *N*-stage. High riskscore is often associated with poorer prognosis, higher T-stage, and *N*-stage. Based on our clinical screening of PSA and blood expression levels of AC007405.2 and AL354989.1, we found that ARR-RSM is a more accurate diagnostic biomarker for PCa. RT-qPCR results revealed that AC007405.2 and AL354989.1 are highly expressed in PCa cells (LNCaP) and clinical PCa tissues.

Previously, multiple high-quality studies have reported the pivotal regulatory role of lncRNAs in PCa. Dysregulation of lncRNA OIP5-AS1 expression could mediate PCa cell growth and cadmium-induced ferroptosis. Mechanistically, OIP5-AS1 served as an endogenous sponge of miR-128-3p to regulate the expression of SLC7A11, a surrogate marker of ferroptosis [31]. LncRNA MEG3 was a down-regulated lncRNA in PCa tissues and cells and could inhibit the expression of miR-9-5p, whereas miR-9-5p down-regulated QKI-5 expression. Overexpressed MEG3 and QKI-5 could decrease the abilities of proliferation, migration and invasion in PCa cells effectively and increased the apoptosis rate [32]. LncRNA DSCAM-AS1 was transcriptionally activated by super-enhancers driven by FOXA1 and exhibited lineage-specific expression pattern. DSCAM-AS1 could promote cancer progression by interacting with YBX1 and regulating expression of FOXA1 and ERα [33]. Yuliang Wang et al. indicated that FRGPI is not only a promising and robust prognostic biomarker but also a potential indicator of immunotherapy outcomes in patients with PCa after radical prostatectomy [34]. At present, researchers have focused on the relationship between lncRNA and AAT resistance. Dong et al. found that the LncRNA SNHG4 promotes PCa cell resistance to ENZ through let-7a/RREB1 positive feedback loop and ceRNA network [35]. Chen et al. found that LncRNA ERVH48-1 activates Wnt/β-catenin pathway through sponging miR-4784, which promotes drug resistance and proliferation of PCa [36]. However, the mechanisms of lncRNAs-mediated AAT resistance remain poorly understood. In our study, we discovered the biological roles of AC007405.2 and AL354989.1 in AAT resistance. SiR-AC007405.2 and siR-AL354989.1 can inhibit the proliferation ability of LNCaP. The qPCR analysis revealed a significant upregulation of AC007405.2 and AL354989.1 in LNCaP cells treated with ENZ, ARN, and BIC. SiR-AC007405.2 and siR-AL354989.1 notably enhanced the apoptotic effects induced by ENZ, ARN, and BIC in LNCaP cells. Recently, it has been reported that the increased JAK/STAT and FGFR signal transduction drive AAT resistance in PCa [37]. Similarly, the results of our KEGG enrichment analysis also suggest that ARR-LncRs is mainly enriched in the JAK/STAT signal pathway, which not only reveals the mechanism of ARR-LncRs, but also further confirms the important role of ARR-LncRs in AAT resistance. In summary, AC007405.2 and AL354989.1 could serve as potential molecular therapeutic targets and enhancers of AAT treatment efficacy in the future.

These findings highlight the role of ARR-RSM in the prognostic assessment of PCa patients and the relationships between ARR-LncRs (AC007405.2 and AL354989.1) and clinical features. By establishing ARR-RSM, it can replace PSA as a new screening indicator for early PCa diagnosis, reducing the mortality and incidence rates of PCa. In addition, we can evaluate the potential risk of AAT treatment resistance in patients before treatment and develop personalized treatment plans based on the predictive results, thereby achieving more accurate medical care. In summary, ARR-RSM is an indispensable tool in clinical practice that can help physicians make more accurate diagnostic and treatment decisions, ultimately improving the quality of life for patients. The use of ARR-RSM to predict the presence of PCa was limited to a single-center cohort. In future studies, we will expand our model to multiple centers to achieve the generalizability of ARR-RSM. Regarding the molecular mechanisms underlying the promotion of PCa proliferation and AAT resistance by AC007405.2 and AL354989.1, we have only conducted KEGG pathway analysis, which identified the JAK-STAT pathway. However, further validation is needed through relevant *in vitro* and *in vivo* experiments.

## CONCLUSIONS

In this study, we have uncovered the clinical significance of AC007405.2 and AL354989.1 in predicting the prognosis and diagnosing patients with PCa. Additionally, we have validated their association with various clinical features. Furthermore, we validated the high expressions of AC007405.2 and AL354989.1 in PCa tissues and cell lines, and demonstrated their ability to promote PCa cell proliferation and resistance to AAT treatment. These findings offer potential targets for the treatment of PCa and provide a novel diagnostic and predictive indicator for accurate PCa diagnosis.

## Supplementary Materials

Supplementary Figure 1

Supplementary Table 1

Supplementary Tables 2 and 3
