# Supplementary material for: AAT resistance-related AC007405.2 and AL354989.1 as novel diagnostic and prognostic markers in prostate cancer
Source: Aging (Albany NY). 2024 Apr 19;16(8):7249–66. doi: 10.18632/aging.205754 (PMC11087092; doi:10.18632/aging.205754)
Supplement: Supplementary Figure 1 [file aging-16-205754-s001.pdf]

SUPPLEMENTARY FIGURE

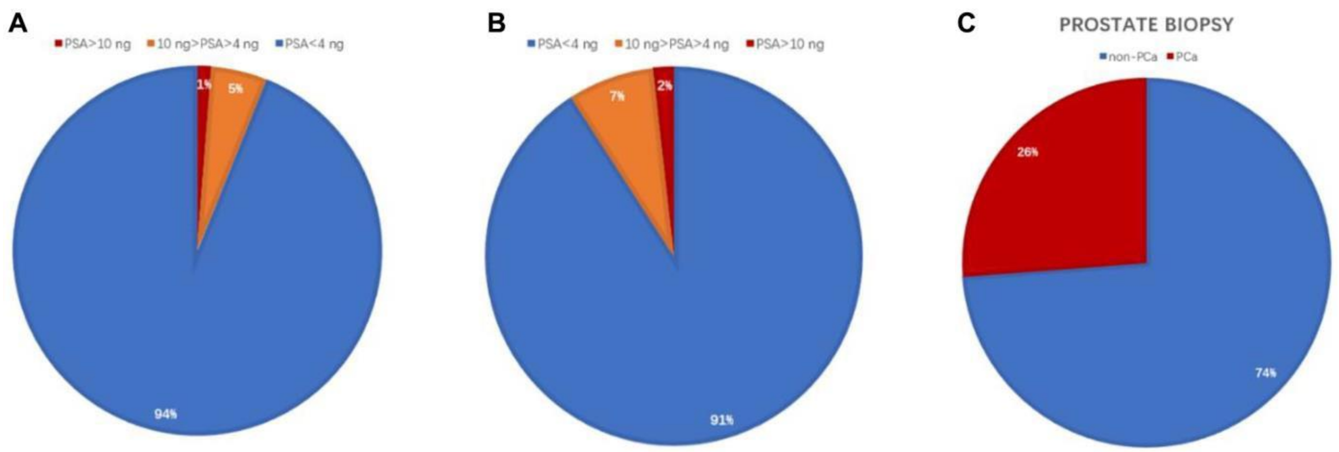

Supplementary Figure 1. (**A**) PSA screening results of 16,746 males in the Sichuan and Chongqing region (blue represents PSA <4 ng/ml, orange represents 4 ng/ml < PSA <10 ng/ml, red represents PSA >10 ng/ml). (**B**) PSA screening results of 570 males in the First Affiliated Hospital of Chongqing Medical University (blue represents PSA <4 ng/ml, orange represents 4 ng/ml < PSA <10 ng/ml, red represents PSA >10 ng/ml). (**C**) Prostate biopsy results of 42 males in the First Affiliated Hospital of Chongqing Medical University with abnormal PSA levels (blue represents no confirmed PCa, red represents confirmed PCa).
