# Supplementary material for: AAT resistance-related AC007405.2 and AL354989.1 as novel diagnostic and prognostic markers in prostate cancer
Source: Aging (Albany NY). 2024 Apr 19;16(8):7249–66. doi: 10.18632/aging.205754 (PMC11087092; doi:10.18632/aging.205754)
Supplement: Supplementary Tables 2 and 3 [file aging-16-205754-s003.pdf]

SUPPLEMENTARY TABLES

Supplementary Table 2. The multivariate Cox analysis of the sARR-LncRs.

Id	coef	HR	HR.95L	HR.95H	*p*-value
AL354989.1	2.037602888	7.672196028	1.453281244	40.5032351	0.016380036
AL391427.1	1.765286435	5.843245828	2.494290438	13.68867125	4.82E-05

Supplementary Table 3. tPSA and AC0070405.2+AL354989.1 value of the First Affiliated Hospital of Chongqing Medical University patients.

Patients	tPSA(ng/mL)	AC007405.2+ AL354989.1 (riskscore)	Diagnosis
1	5.41	0.766024194	non-PCa
2	5.74	0.778230729	non-PCa
3	8.41	0.813536458	non-PCa
4	8.23	0.88687108	non-PCa
5	4.86	1.001053169	non-PCa
6	8.03	1.013259705	non-PCa
7	7.48	1.466883259	PCa
8	6.93	1.131383426	non-PCa
9	8.63	1.508949194	PCa
10	6.12	1.183028142	non-PCa
11	5.88	1.172040074	non-PCa
12	4.99	1.274111038	non-PCa
13	6.16	1.252325722	non-PCa
14	4.59	1.396367212	non-PCa
15	4.85	1.351291882	non-PCa
16	8.77	1.394862514	non-PCa
17	7.43	1.366221582	non-PCa
18	6.26	1.439747024	non-PCa
19	5.95	1.445097943	non-PCa
20	8.02	1.424626504	non-PCa
21	9.21	1.498151946	non-PCa
22	6.88	1.487068467	non-PCa
23	5.5	1.593176474	PCa
24	7.25	1.708672441	non-PCa
25	8.19	1.660969357	non-PCa
26	5.77	1.792995131	non-PCa
27	9.05	1.746605924	non-PCa
28	8.08	1.833747189	non-PCa
29	6.7	1.855437095	PCa
30	7.37	1.881164043	non-PCa
31	6.04	1.885201085	non-PCa
32	5.59	1.939664375	non-PCa
33	15.9	1.929990184	non-PCa
34	38.95	1.967923667	non-PCa
35	37.99	2.075440961	non-PCa
36	39.44	2.026328589	PCa
37	39.58	2.152812624	PCa

38	19.29	2.10510954	PCa
39	45.88	2.147270885	PCa
40	49.44	2.156754256	PCa
41	56.8	2.35779138	PCa
42	68.62	2.410749973	PCa
